# Evidence for Culturally Used Herbal Medicines with Psychiatric Benefits in the Paediatric Population: An Evidence Map to Inform a Systematic Review

**DOI:** 10.1192/bjo.2026.11118

**Published:** 2026-06-30

**Authors:** Jiayang Han, Mallika Punukollu

**Affiliations:** 1 University of Edinburgh, Edinburgh, United Kingdom; 2 University of Glasgow, Glasgow, United Kingdom

## Abstract

**Aims:**

In seeking treatment for children presenting with psychiatric symptoms, many families ask about use of complementary and alternative medicine (CAM), including herbal preparations, to help their child cope with mental health distress in addition or alongsideconventional care. This includes remedies to support their child through anxiety, sleep disturbance, low mood and attention difficulties. In UK paediatric populations, CAM use is widespread (41% in one tertiary survey), and 44.6% of families managing childhood ADHD report non-mainstream treatments. However, clinical evidence of their efficacy and tolerability is weak and rarely synthesised.

In today’s multidisciplinary healthcare system, an understanding of CAMs, including their psychiatric outcomes and safety sensitivity, will improve shared family decision-making in child and adolescentpsychiatry. Our aim is to map paediatric interventional and published trial evidence for 19 culturally used botanicals shortlisted for reputed psychiatric/behavioural benefits in children to be included in a systematic review.

We hypothesised the clinical literature would be sparse, heterogeneous, and majorly consisting of smaller studies.

**Methods:**

Nineteen botanicals were shortlisted from commonly used traditional preparations with plausible tolerability in children and reputed benefits for psychiatric/behavioural symptoms. We conducted an evidence map of these herbs using two sources: (1) ClinicalTrials.gov (“Find Studies”) filtered to interventional studies, age group “child”, and status “completed”; and (2) PubMed searches using herb name/botanical name terms combined with child/adolescent terms and publication types “clinical trial” or “randomized controlled trial”. For each herb, we recorded trail counts and manually screened each record’s primary outcome/condition to retain only those with psychiatric/behavioural relevance. This includes anxiety, depression, ADHD (Attention-Deficit/Hyperactivity Disorder), sleep, mood, and cognition.

**Results:**

ClinicalTrials.gov identified 52 completed paediatric interventional records across11/19 herbs (median 1 per herb; range 0–19). Counts were highly skewed: lavender (19) and chamomile (8) accounted for over half of registry records; eight herbs had no completed paediatric interventional record. PubMed trial-type searches retrieved 36 publications across 11/19 herbs (median 1; range 0–9), similarly concentrated: lavender (9), moringa (8) and chamomile (7) comprise two-thirds of trial-type publications. Across both registries, studies had variable measurements of psychiatric/behavioural outcomes, and inconsistent reporting of adverse effects.

**Conclusion:**

Paediatric interventional and published trial evidence for culturallyused botanicals with reputed psychiatric benefits is limited and unevenly distributed. Many candidates are supported by zero or single trials and heterogeneous outcomes. This evidence map will be used to inform a focused systematic review investigating mental health outcomes and tolerability and identify best candidates for further research.

